# Tracking of voluntary exercise behaviour over the lifespan

**DOI:** 10.1186/s12966-019-0779-4

**Published:** 2019-02-04

**Authors:** Matthijs D. van der Zee, Denise van der Mee, Meike Bartels, Eco J. C. de Geus

**Affiliations:** Department of Biological Psychology, Netherlands Twin Register, Vrije Universiteit Amsterdam, The Netherlands, Van der Boechorststraat 7, 1081 BT Amsterdam, The Netherlands

**Keywords:** Longitudinal stability, Behavioural trends, Leisure time physical activity, Lifespan, Competitive exercise, Team exercise

## Abstract

**Background:**

The aim of many physical activity interventions is to develop life-long habits of regular exercise and sports activities in leisure time. Previous studies that assessed tracking (i.e. the stability of a trait over the lifespan) of leisure time exercise behaviour across various parts of the life span have treated it as a uniform construct by summing all types of leisure time exercise activities into a single summary score for the total volume of exercise. This study provides new insight by additionally determining tracking across leisure time exercise activities in six different domains: (1) team-based versus solitary activities, (2) competitive versus non-competitive activities, and (3) externally paced versus internally paced activities. We also assessed which of the domains of exercise activities best predicted total volume of exercise at follow-up.

**Methods:**

A large dataset (*N* = 43,889) from the Netherlands Twin Register (NTR) was used to analyse the tracking of exercise behaviour over time. Using this dataset, we were able to examine tracking as a function of baseline age (8 to 80 years) and tracking duration (2 to 22-year follow-up), taking into account sex differences, using generalized estimating equations.

**Results:**

Two-year tracking coefficients are moderate to high for total volume of exercise across ages at baseline, ranging from .38 to .77 with a median of .57. Tracking coefficients tend to decrease as the distance to follow-up increases, down to a median of .38 for the 22-year tracking coefficients. The patterns of tracking were largely domain-independent and were largely similar for solitary, competitive, non-competitive, externally and internally paced activities. With the exception of team-based activities, tracking was seen to increase as a function of baseline age. Cross-domain tracking did not favour any specific domain of exercise activity as the best predictor for total volume of exercise behaviour and this was true at all baseline ages.

**Conclusion:**

We conclude that exercise behaviour is moderately to highly stable across the life span. In particular in adulthood, where the tracking of exercise mimics that of a classical behavioural trait like personality. This stability reinforces existing evidence that exercise habits are hard to change, but at the same time suggests that successful intervention leading to the adoption of exercise habits will tend to last.

**Electronic supplementary material:**

The online version of this article (10.1186/s12966-019-0779-4) contains supplementary material, which is available to authorized users.

## Background

In our evolutionary history, the physically demanding environment caused genes in humans to be specifically selected to cope with high levels of physical activity. This resulted in human physiology in which most systems (e.g. metabolic and cardio-vascular) do not function optimally unless regularly engaged by sufficient physical activity [1]. However, technological advancements, made since the industrial era, have continually decreased the necessity of physical activity, both in work- and home-environments. This has drastically reduced physical hardships of workers as well as the amount of disabilities caused by jobs that demand heavy labour, but unfortunately these beneficial aspects of lower levels of physical activity are mirrored by detrimental effects on the risk for chronic non-communicable diseases. Physical inactivity is considered a well-established risk factor for cardiovascular disease and cancer [2, 3] as well as for chronic psychiatric disorders [4]. The importance of intervention on physical activity has been stressed for many decades [5, 6].

Such interventions can be aimed at increasing total physical activity including means of transportation and work-related physical activity, or they can specifically target leisure time physical activity. A disadvantage of targeting total physical activity is that it can be highly dependent upon factors that are outside of a person’s immediate control, such as the type of employment, and the mode of travel to and from work allowed by the distance between home and work locations. Workplace based exercise facilities are offered by some but not by all employers, and not all allow this to be done in office hours. From a public health perspective, increasing voluntary physical activity during leisure time may be a more viable target for behavioural intervention. Such interventions might be well served by building on the natural tendency to participate in sports and exercise activities that is still evident in many people. Such tendencies are actively nursed by physical education classes in primary and secondary education and by public health policies supporting sports and exercise in youngsters by co-financing sports clubs and facilities. However, the extent to which this leads to the development of life-long habits of regular exercise behaviour in leisure time remains to be established. This is unfortunate because the physiological and psychological benefits of regular physical activity do not seem to last if such activity is discontinued [7, 8].

A number of ‘tracking’ studies have been performed to identify patterns in the development of leisure time physical activity over the lifespan. Tracking is typically defined as the tendency of individuals to maintain their rank or position within a group over time [9] and tracking coefficients of sufficient magnitude allow a reliable prediction of future exercise behaviour from the current behaviour. Not surprisingly, most research on tracking of leisure time exercise behaviour has been focused on tracking from childhood to adolescence [10–13], or on tracking in the transition from childhood/adolescence to adulthood [7, 14–18]; although some studies have also looked at tracking in middle-aged and older cohorts [15, 19–23]. A number of replicated results can be extracted from these studies [9, 24]. First, tracking of leisure time physical activity decreases with the duration of follow-up with tracking coefficients from baseline to follow-up of 0.37 to 0.71 on shorter intervals of three to 9 years, but lower tracking when time intervals are longer than 9 years, as low as 0.07 in some cases of 31-year follow-ups [15]. Second, tracking from childhood to adolescence or from adolescence to adulthood is lower than the tracking across the longer phase of adulthood itself. Third, in keeping with the first two findings, lowest tracking is found from childhood to adulthood. Fourth, tracking seems to be influenced by sex, with lower coefficients found in women than in men [9].

A major shortcoming of all these previous studies is that they have treated leisure time exercise behaviour as a uniform construct by summing all exercise and sports activities that the person engages in into a summary score [15, 25] that either reflects the time spent on exercising, or an estimation of the total energy expended in exercise. The latter is done by multiplying frequency, duration, and an estimate of the intensity of the exercise activities usually expressed as a multiple of the basal metabolic rate (i.e. in MET hours weekly). Although the use of such a summary score is very valuable, it cannot detect more detailed patterns in the tracking of different types of exercise behaviour. Ideally tracking coefficients would be analysed for each sport or exercise activity individually (fitness, soccer, jogging, tennis, karate, etc.) but doing so would significantly reduce the number of available subjects per cell even in very large databases. Therefore, as a compromise, exercise activities could be divided into domains based on three dimensions [1] a team-based or individual nature, [2] a competitive or non-competitive nature, and [3] an externally paced or an internally paced nature. The third of these dimensions is based on the nature of the skills required in the exercise activities [26, 27]. These domains were chosen because exercise behaviour in these different domains of exercise behaviour is likely to be differently sensitive to the many transitions and life-changing events experienced during the course of life. Their contribution to the overall volume of exercise and sports behaviour may likewise differ across time. Team-based exercise behaviour for example is expected to track less well into adulthood when compared to solitary activities [28, 29], likewise participation in competitive exercise is expected to decline [30]. We believe personality and cognitive flexibility to be the major determinants of a preference for internally, or externally paced exercise [27] and thus, given the stability of personality, produce larger tracking in these domains. A better understanding of the development of life-long habits of regular exercise behaviour in these various domains, and particularly the identification of ‘vulnerable periods’ with lowered tracking is directly needed. Supporting such a favourable development still proves a major challenge in many societies.

Using a large nationwide dataset (longitudinal *N* = 43,889), available at the Netherlands Twin Register (NTR), our aim is to assess the shortcomings mentioned above, allowing us to add significantly to the existing knowledge of the longitudinal tracking of exercise behaviour in various domains. The NTR dataset spans a wide age range and includes children, adolescents and adults (8–82 years) in which exercise behaviour has been assessed longitudinally in a very similar manner in biennial NTR survey waves. This unique resource allows us to analyse tracking-coefficients of up to a 22-year follow-up, to assess this long-term tracking with starting baseline ages in childhood, adolescence or adulthood, and to split all analyses by sex to compare tracking coefficients in males and females. Importantly, the information provided by the subjects allowed us to compute the tracking coefficients across total MET-minutes weekly but also separately for MET-minutes in the domains of team vs individual, competitive vs non-competitive, and externally paced vs internally paced exercise activities. We expected to find significant stability for the leisure time exercise behaviours in all domains with higher tracking coefficients across shorter time spans, lower values in females than in males, and a gradual increase in tracking from childhood to adulthood.

## Methods

### Participants

The NTR is an ongoing research initiative that includes twins and their relatives. Subjects (both adults and children) are assessed every two to 3 years using extensive surveys. Subjects aged 14 and older fill out self-report surveys, and parents of children under 14 are asked to fill out surveys on the behaviour of their children. In all cases the parent-rating of the mother is preferred and if this rating is not available, rating of the father is used (inter-rater correlations ranged from .74 to .85 depending on the age of the subject). Methods and procedures used for data collection in the NTR are described elsewhere [31, 32]. Subjects were included only if any measure of voluntary exercise behaviour was available in at least two surveys (*N* = 43,889).

### Voluntary exercise behaviour

Overall physical activity has proven to be difficult to measure reliably through self-report [33], but the more salient regular exercise activities voluntarily done in leisure time are much more reliably recalled [34, 35]. The current study focusses on voluntary regular exercise behaviour in leisure time, collected by virtually the same survey methods in large numbers of twins, their parents, siblings, spouses and the children of twins and siblings [31, 32]. To assess the volume of voluntary exercise behaviour we ask whether the participant exercised regularly in their leisure time (‘Yes’ or ‘No’). If the participants (or their parental informant) responded affirmative, they were asked to indicate all of the exercise activities done regularly. For each exercise activity reported, they noted the number of months per year, weekly frequency and average duration in minutes of the activity. Depending on the age of the participants (≤16 vs > 16) Ainsworth’s Compendium of Physical Activity [36] or the adaptation for children by Ridley [37] was used to assign a Metabolic equivalent of task (MET) value to each exercise activity, reflecting its energy expenditure as a multiple of the basal energy expenditure (approximately 1 kcal/kg/hour) in an average subject engaged in that activity. For each participant, a total weekly MET value (METmin) was computed across all exercise activities by summing the products of the number of minutes spent weekly on each exercise activity and its MET value. Activities were only considered if that participant had engaged in them for at least 3 months during the past year, thereby excluding short-lived, non-regular activities, such as outdoor skiing in winter or beach sports in summer holidays. For children and adolescents, exercise during obligatory physical education classes at school was not included in the weekly MET value for voluntary exercise behaviour.

Six additional variables were computed based on the specific type of exercise and sports activities. The first was the total volume spent on competitive exercise activities, i.e. activities where an individual, or team, has to compete against another individual, or team for victory. If a subject’s only voluntary exercise behaviour was spent in non-competitive sports (e.g. cardio, muscle training) this was counted as zero METmin (as was done for all non-exercisers). Conversely all METmin spent on non-competitive activities were also summed into a second variable for total volume spent on non-competitive exercise activities. The third variable was the sum of all METmin spent on team-related activities, in which exercisers work together to achieve a common goal. Conversely, all of the METmin spent on solitary exercise (e.g. horse sports, aiming based sports or fighting sports) was summed to a fourth variable. The fifth and sixth variable were based on the exercise being either externally paced (exercise activities where the environment controls the rate of performing the activity) or internally paced (exercise activities where the performer controls the rate at which the activity is performed).

### Statistical analyses

Sex-differences, age effects, age-by-sex interaction, and the quadratic age effect on exercise behaviour were analysed in a cross-sectional dataset containing the last non-missing observation of each subject. These effects were fitted using generalized equation estimation (GEE) models, in order to control for the nested (family) structure of the data. In the GEE model the correlation structure was set to be exchangeable as suggested for family data by previous work [38].

Because surveys were generally sent out every 2 years, ages were transformed to always be even for the tracking analyses. Distance from age at baseline to age at follow-up were therefore always multiples of 2 years, the longest follow-up duration being 22 years. The tracking of exercise behaviour was computed for every combination of baseline age and follow-up duration (i.e. 2-year, 4-year, 6-year, .., 20-year, 22-year) where at least 50 complete combinations were available. GEE models were used to correct for family structure that was present in the NTR, as mentioned earlier. A GEE model was fitted with family as a random factor, and exercise at follow-up as dependent variable. Before the GEE slope-estimates were estimated, MET-scores at baseline and follow-up were normalized. This normalization allows us to consider the slope-estimates as tracking-coefficients, enabling a comparison of our results to results from previous publications where a correlation was used. The standardized slope estimates were plotted in a heatmap that represents tracking as a function of the age at baseline and the follow-up duration.

To analyse the changes in exercise tracking across the lifespan, N-weighted linear regression was performed, regressing the tracking-coefficient on age at baseline for each follow-up duration. This regression was repeated for team-based, individual, competitive, non-competitive, and externally paced or internally paced MET-scores for all subjects together, as well as separately for males and females. To analyse the effects of sex on the tracking-coefficients this linear regression was also run including sex as a predictor in this regression, as well as an interaction effect between sex and age. Because this analysis is repeated over seven exercise domains a *p*-value threshold of .05/7 (0.007) will be considered for statistical significance.

## Results

**Fig. 1:**
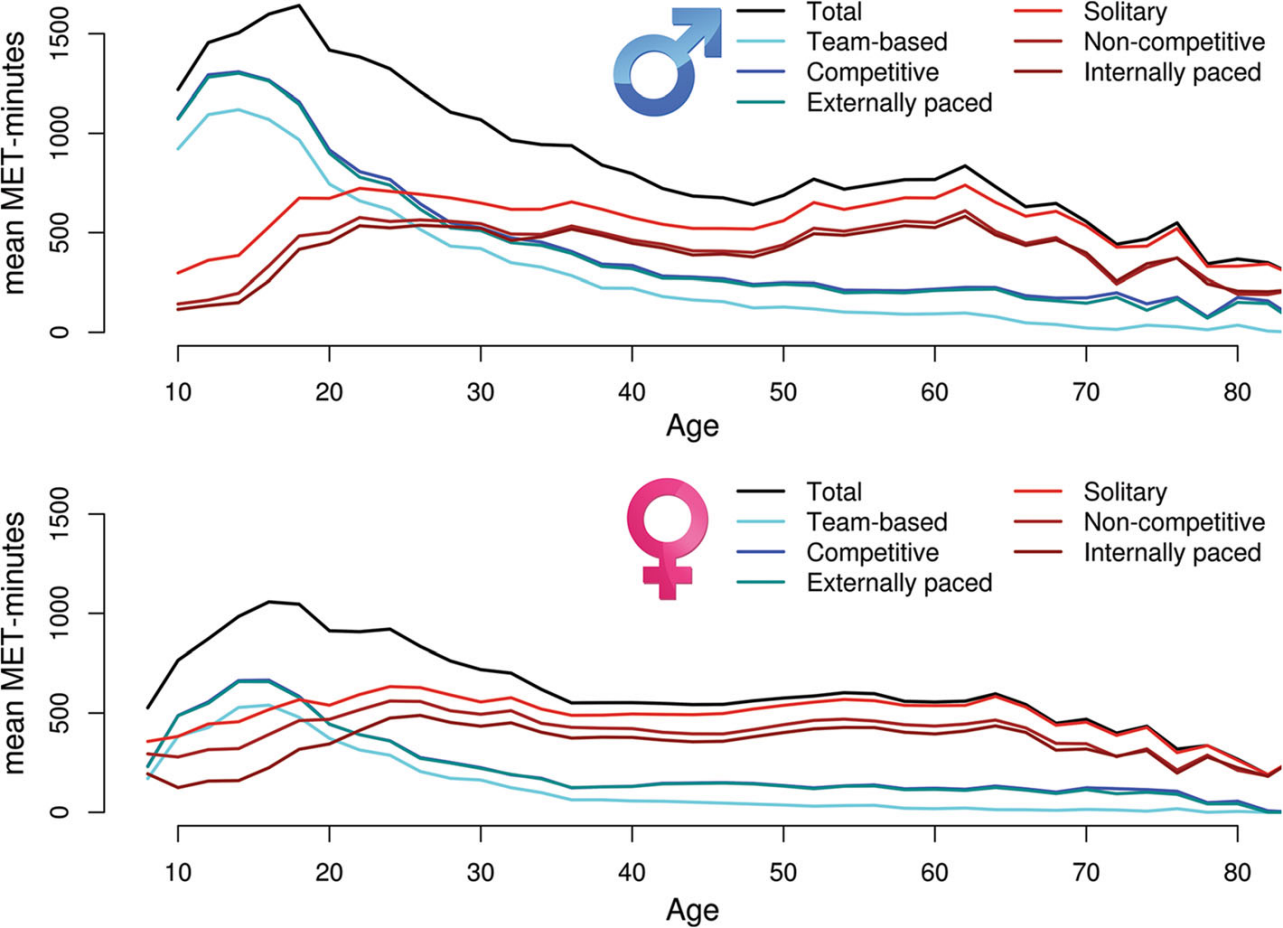
Mean volume of exercise in males (top panel), and females (bottom panel) over time, total volume and volume of exercise in all three dimensions (competitive & non-competitive, team & solitary, and externally & internally paced)

Means and standard deviations for METmin scores in 2-year age-bins are presented in an additional table (see Additional file 1), for the entire sample and graphically displayed separately for males and females in Fig. 1. An increase is seen from age 8 to age 16 for total METmin after which a rapid decline sets in that gradually slows down in mid adulthood. The major source of this biphasic pattern is participation in competitive, team-based, and often externally paced activities that is characteristic of Dutch children and adolescents and includes the most endorsed sports soccer and field hockey [39]. In particular team participation drops sharply after age 16–18, reaching low levels (< 40 METmin weekly) after age 50. A contrasting pattern is seen for solitary non- competitive and internally paced activities which gradually increase to a peak level in early adulthood staying reasonably constant till age 50, after which they also decline. The most endorsed solitary exercise activities were running and weight/muscle training in fitness centres.

Formal testing of the effects of age and sex on METmin in a GEE model revealed significant linear decrease (*β* = − 26.8, *p* < 10^− 90^) of exercise over age, a significant quadratic effect (*β* = 0.04, *p* = 0.0006), as well as a significant (*p* < 10^− 90^) effect of sex with males showing higher levels of exercise. Additionally, the GEE model revealed a significant (*p* = 5.31*10^− 65^) interaction effect between age and sex demonstrating that the decrease of exercise over time is less pronounced in females when compared to males. The age and sex effects are consistent across total volume, competitive METmin, team-based METmin and externally paced METmin. For solitary, and non-competitive METmin females had higher values than males (*p* < 10^− 90^, and *p* = 2.55*10^− 31^ respectively), while for internally paced METmin the effect of sex was not significant (*p* = 0.86). For solitary (*β* = 19.22, *p* < 10^− 90^), non-competitive (*β* = 22.67, *p* < 10^− 90^), and internally paced (*β* = 22.78, *p* < 10^− 90^) METmin a positive linear effect of age is observed, a negative quadratic effect (*p* < 10^− 90^ in all cases), and a negative age-by-sex interaction term in these domains (*p*_*solitary*_ = 1.73*10^− 22^, *p*_*non-competitive*_ = 4.12*10^− 39^, *p*_*internally paced*_ = 1.98*10^− 11^) shows that the increase in exercise over time is less pronounced in females compared to males.

**Fig. 2:**
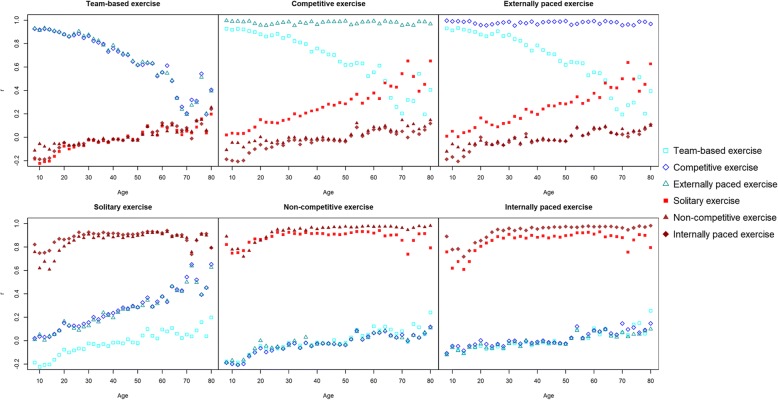
Correlations (r) between exercise domains as a function of age

Correlations at various ages (8 to 80) of exercise activities in the various domains are presented in Fig. 2. It is clear that internally paced, non-competitive, and solitary exercise correlate highly at all ages, and that competitive and externally paced exercise also correlate highly at all ages. For team-based exercise a more complex pattern is seen. It correlates highly with externally paced and competitive exercise in childhood and adolescence, but this correlation decreases strongly in adulthood. In parallel, the correlation of externally paced and competitive exercise with solitary exercise increases across adulthood.

### Tracking

**Fig. 3:**
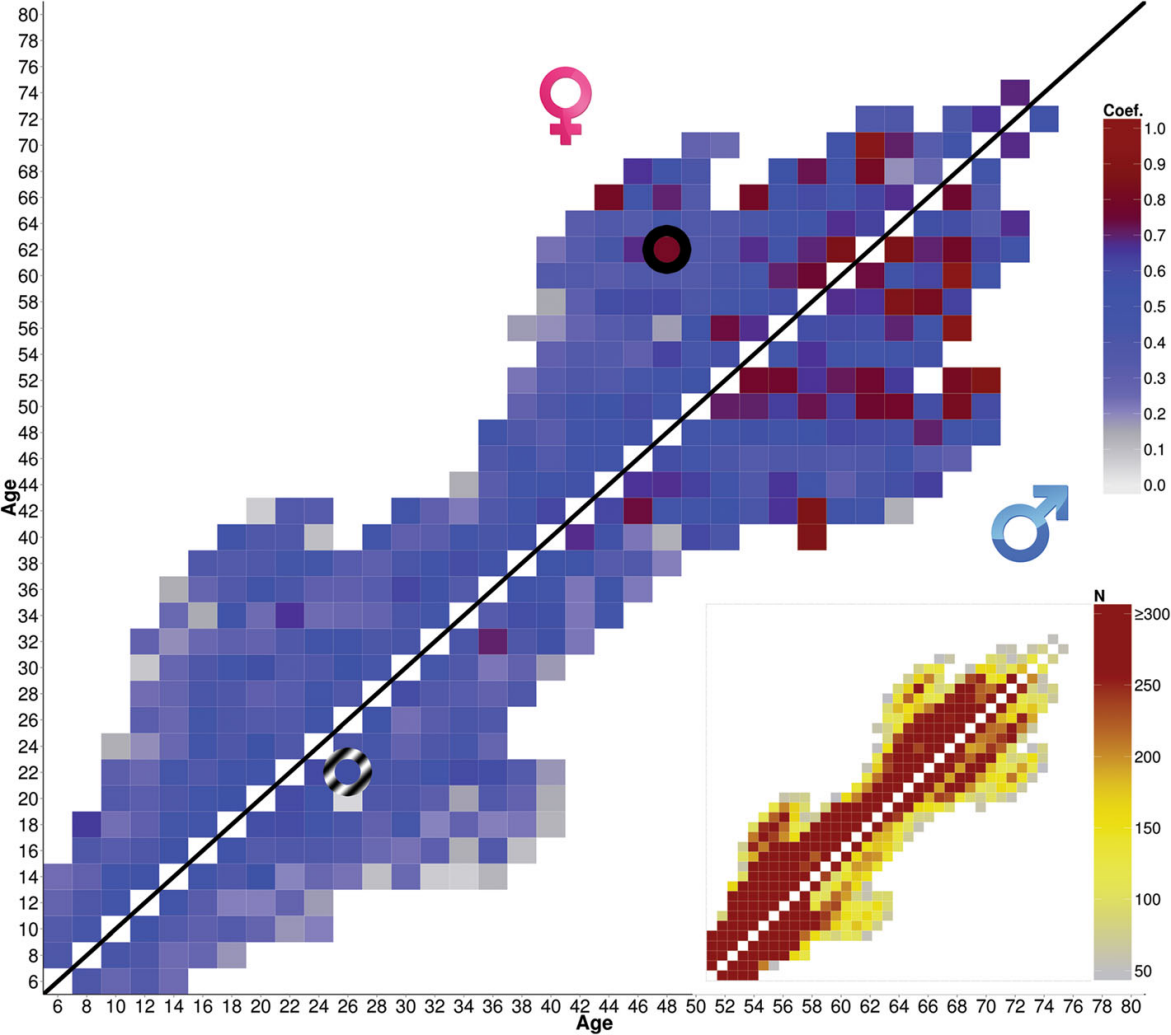
Tracking coefficient heatmap of total volume of exercise. The inset in the bottom right corner represents sample size (N) in each cell, cut off at *N* = 300 for visual purposes. Colours in the main panel represent the value of the tracking coefficient (grey to blue to red from low to high), colours in the inset represent number of samples (grey to yellow to red from low to high). To demonstrate; the cell in the full circle represents the tracking coefficient (0.81) of age 48 at baseline, and age 62 at follow-up in females. The cell in the striped circle represents the tracking coefficient (0.57) of age 22 at baseline, and age 26 at follow-up in males

All available tracking coefficients split by males and females for total METmin are displayed in the heatmap in Fig. 3 and tracking coefficients for the various domains are displayed in an additional Figure (see Additional file 2). Insets show the sample size for each of the heatmap entries (all coefficient were based on *N* > 50). A summary of 2-, 8-, 14-, and 20-year tracking coefficients of all domains is provided in an additional table (see Additional file 3).

Two-year tracking coefficients are moderate to high for total METmin across all ages, ranging from .38 to .77 with a median of .57. Tracking coefficients tend to decrease as the distance to follow-up increases, ranging from a median of .57 at 2-year tracking down to a median of .38 for 22-year tracking coefficients. A similar pattern of decreased tracking with increased follow-up duration is seen for all other exercise domains (see Fig. 3).

### Changes in exercise tracking across the life span

**Fig. 4:**
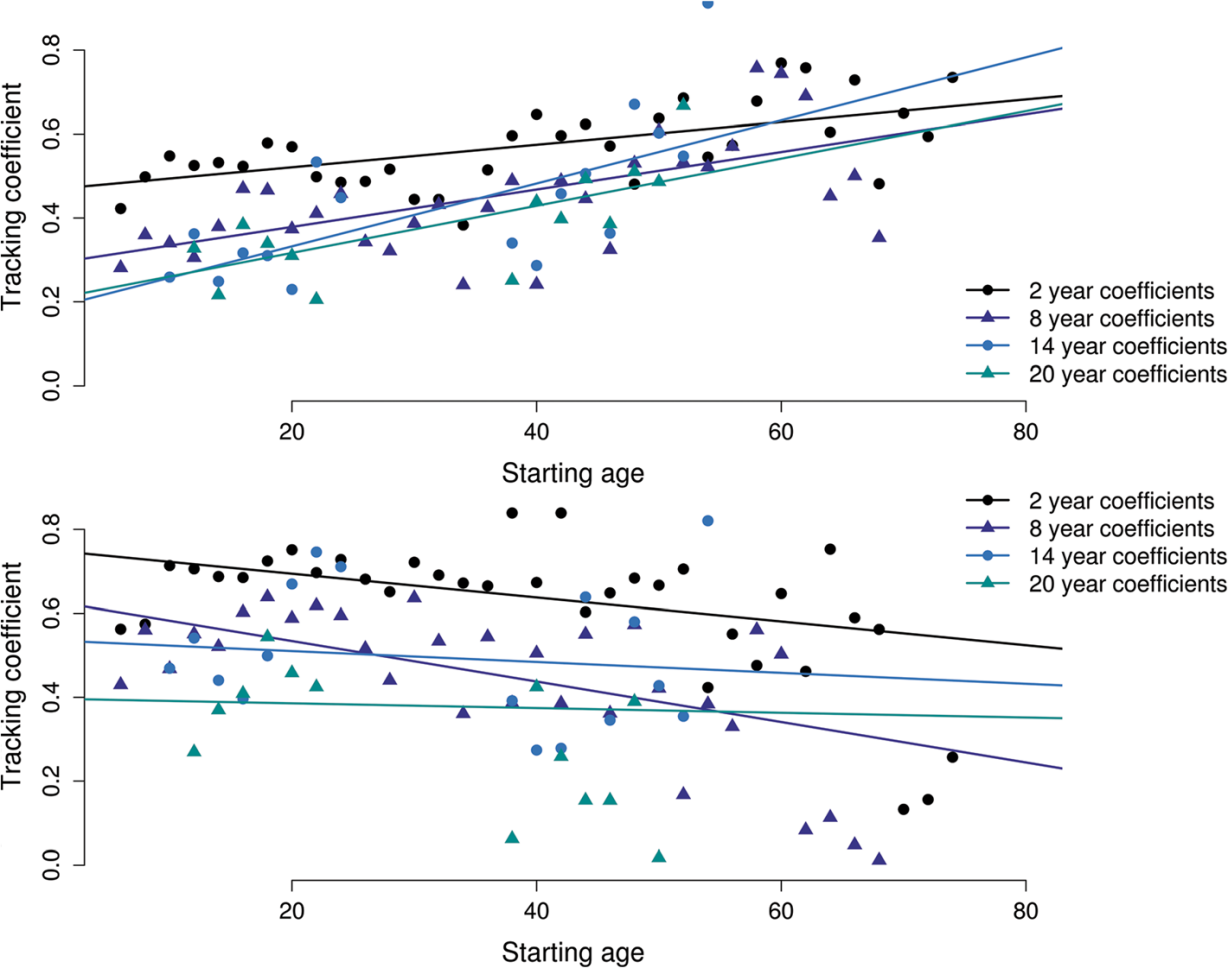
Tracking coefficients for total volume of exercise behaviour (top panel), and team-based exercise (bottom panel) as a function of age at baseline

The tracking of exercise as expressed by total METmin shows a clear increase with age at baseline regardless of distance to follow-up. Figure 4 fits regression lines to the tracking-coefficients for follow-up durations 2, 8, 14 and 20 years as a function of the age at baseline. The increase in tracking across the life span held for all other exercise subdomains (for plots see Additional files 4, 5, 6, 7 and 8) with the single exception of team-based METmin where tracking coefficients often decrease with age (see Fig. 4).

It is important to observe that, regardless of which type of exercise is analysed, there is no noticeable change in the patterns of tracking coefficients when the informant changes, that is when we change from parent-rated to self-rated voluntary exercise behaviour (from 12 to 14 years) with the same instrument.

### Sex differences in tracking

To assess sex-differences in the tracking-coefficients the previous linear regression model was expanded to include an effect of sex, and an the interaction of sex and age. Significant main effects of sex were present in total METmin and competitive METmin where tracking was slightly higher in females compared to males. In total METmin, as well as solitary, non-competitive and internally paced METmin the interaction was also significant, suggesting that in these domains the increase of the tracking coefficient over time is less pronounced in females compared to males.

### Cross-domain tracking

In an exploratory analysis we tested whether team-based, individual, competitive, non-competitive, externally paced or internally paced activities were the best predictor of the total volume of exercise at follow-up. For this, the above analyses were repeated with METmin in any of the domains at baseline, and the total amount of MET-minutes spent exercising at follow-up in the GEE models described earlier.

Heatmaps of tracking coefficients of any exercise domain at baseline are displayed in an additional figure (see Additional file 9), and the summary of 2-, 8-, 14-, and 20-year tracking coefficients of all domains is included in an additional table (see Additional file 2). Overall, the cross-domain tracking coefficients were lower than the within-domain tracking coefficients. For team-based the median two-year tracking coefficients was 0.24, compared a median of 0.67 for within-domain tracking coefficients. For competitive, and non-competitive exercise the median two-year cross-domain coefficients were 0.35 and 0.41 compared to 0.67 and 0.50 within-domain respectively. For externally paced and internally paced exercise the median two-year cross-domain coefficients were 0.35 and 0.41 compared to 0.66 and 0.52 within-domain respectively.

Only for solitary based exercise were the median two-year cross-domain tracking coefficients (0.49) very comparable to that of within-domain tracking (0.52). The magnitude of between-domain tracking decreased as the distance to follow-up increased, just as was seen for within-domain tracking. This led to a decrease in the differences between within-domain and between domain tracking, because the ‘surviving’ exercise activity had become the main source of the total volume of exercise behaviour. For team-based exercise, the median 22-year cross-domain tracking coefficient of team-based exercise was 0.21 compared to 0.27 in within-domain coefficients. For solitary based exercise the median 22-year tracking between-domain coefficient was 0.17 compared to 0.21 within-domain. For competitive and non-competitive the median 22-year cross-domain coefficients were 0.24 and 0.19 compared to 0.28 and 0.20 within-domain respectively. For externally paced and internally paced exercise the median 22-year cross-domain tracking coefficients were 0.24 and 0.21 compared to 0.27 and 0.29 within-domain respectively.

Similar to what was done for the within-domain coefficients, linear regression of the tracking coefficients on age at baseline was performed across different follow-up durations. Regressions for 2-, 8-, 14-, and 20-year coefficients for each domain are given in additional figures (see Additional files 10, 11, 12, 13, 14 and 15). These slopes show the pattern that was already obvious from Fig. 1: the total volume of exercise behaviour in METmin is best predicted by previous levels of competitive, team-based activities in childhood and adolescence, but much better by non-competitive, solitary, and internally paced exercise after age 30.

## Discussion

The aim of this paper was to examine tracking of voluntary exercise behaviour in leisure time over the entire lifespan. Exercise activities were clustered in different domains reflecting the nature of the activity to be [1] competitive or non-competitive, [2] team or solitary, and [3] externally or internally paced. The prevalence of engaging in voluntary exercise behaviour, especially competitive, team-based, and externally paced sports increases during childhood, to reach its peak during mid-adolescence, around age 16. After mid-adolescence the total volume of exercise behaviour starts a decelerating downward trend which is largely explained by the decline in participation in competitive, team-based sports. The participation in non-competitive, solitary sports increases from childhood to late adolescence to remain relatively stable between 18 and 64 years (51 to 60% exercisers). After the age of 64 the participation in non-competitive solitary sports starts to decline as well, causing the total participation in regular exercise to drop down to 23.1% at age 82, at which point 100% of the exercise activities engaged in are solitary.

The results of the analysis of tracking-coefficients suggest that tracking of voluntary exercise behaviour is moderate to high (.38 to .57) regardless of the exact domain of exercise activities analysed. In concordance with the expectations from the extant literature, as briefly reviewed in the introduction, tracking coefficients decreased with increased duration to follow-up and were generally lower from childhood or adolescence into early adulthood compared to tracking across early to late adulthood. In deviation of the latter, the tracking coefficients of team-based exercise decreases with age. These findings are in line with previous findings [28, 29]. Contrary to our expectation, competitive exercise did not demonstrate a decline in tracking as substantial as the decline observed in team-based exercise. Given the strong decrease in team sports after adolescence it becomes increasingly difficult to form teams of similar skill and motivation, and [2] may cause some teams to fall apart and exercisers to quit due to a lack of likeminded team-members.

In spite of decreased tracking of team sports, our results show that the tracking coefficients of competitive and externally paced exercise increase, and that their correlation to solitary exercise also increases over time. This suggests that subjects switch from a team-based, externally paced, competitive exercise (such as football or field-hockey) to a solitary, externally paced competitive exercise (such as tennis or squash) later in life. In accordance with previous reports, we observed higher tracking coefficients in males for total volume of exercise, however our results suggest that when baseline age, and an age-sex interaction are taken into account it appears that in total volume, as well as solitary, non-competitive and internally paced exercise the effect of sex is dependent on age at baseline.

We show that long-term tracking from childhood to adulthood for leisure time exercise activities in all domains, albeit significant, is at most modest. This may not be surprising because this period contains some of the major transitional phases and life-changing events that can impact on the time available for leisure exercise, including entering high school, leaving home, taking up a job, and starting a family. Yet, the idea that childhood and adolescence should be specifically targeted to ensure life-long exercise habits is widespread, and motivates physical education classes in primary and secondary education and national, regional, and local policies to enable and encourage sports and exercise in youngsters. Telama [9] formulated a number of mechanisms that could underlie tracking from childhood to adulthood, the carry-over value hypothesis, the ability and readiness hypothesis, the habit formation hypothesis, and the self-selection hypothesis.

The carry-over value hypothesis suggests that early adoption of specific exercise/motor skills acquired in childhood will lead adults to participate in that same exercise. Splitting the analyses over the domains and comparing the results of within-domain tracking coefficients with cross-domain tracking coefficients across the lifespan allows us to address this hypothesis. The carry-over value would predict higher within-domain tracking compared to cross-domain tracking. The ability and readiness hypothesis suggests that subjects with high level of exercise in youth will also be among the higher exercisers in adulthood, regardless of the type of sport. The ability and readiness hypothesis would predict high tracking coefficients from any domain at baseline to total exercise at follow-up. In our data, the ability and carry-over value hypothesis finds stronger support than the ability and readiness hypothesis. Within-domain coefficients were higher than cross-domain tracking coefficients. However, some evidence for the ability and readiness hypothesis is found in late adulthood where the tracking coefficients of individual exercise at baseline and total exercise at follow-up approach those of within-domain total exercise. We caution that this may be a result of (nearly) all exercisers partaking in individual exercise at this stage of life.

The habit formation hypothesis suggests that simply repeating a behaviour many times (independent of skill level) will form a habit. This hypothesis would, more strongly than the carry-over value hypothesis, predict higher within-domain than cross-domain tracking across the entire life span. Our results only partly support the habit formation hypothesis, because substantial exchange between exercise activities across the different domains is seen, although in late adulthood tracking of domain-specific exercise is very high. It may be that habits are more easily formed at later ages due to circumstances allowing for easier habit formation such as a steady job, whereas in childhood, adolescence and young adulthood skill level is more important and there are many other factors impacting on exercise choice (puberty, education, career change, etc.).

Finally, the self-selection hypothesis states that subjects with a genetic predisposition for high exercise ability and/or exercise motivation will more often partake in exercise [40]. Using the twin-family structure of part of the data presented here, we have previously provided evidence for this hypothesis by showing substantial heritability of voluntary exercise behaviour across the life span [41, 42]. However, heritability has been established only for total volume of exercise. For exercise activities in the team-based/solitary, competitive/non-competitive and external/internal paced domains the self-selection hypothesis has not been addressed although a candidate gene study provides some indication of possible different genetic architectures for these domains [27].

A limitation of this study is the use of self-report which may suffer from both recall and social desirability biases. Self-report of participation in sports and leisure exercise time behaviour has shown to be more reliable than self-report on other leisure time physical activities [9]. Whether leisure time exercise behaviour could be better quantified by using objective methods like accelerometers is not an easy question. When the focus is on energy expenditure in leisure time more reliable estimates will likely derive from accelerometers than from self-report. Accelerometer-derived activity counts can, however, not be used to discriminate between competitive and non-competitive or team-based versus solitary activities, at least not without additional self-report on the content of the activities. Accelerometry, typically with a 7-day recording time, may also miss sports activities performed with a frequency that is less than weekly. So far, direct comparison between objective and recall methods have not revealed clear differences in tracking coefficients for leisure time physical activity [9] and self-reported leisure time physical activity robustly predicted objectively measured moderate to vigorous activity across a 25 year follow-up period [43]. Without discrediting recall and social desirability bias, the use of self-report instead of objective measurements may not be overly problematic for these specific behaviours.

## Conclusions

In conclusion, voluntary exercise behaviour in leisure time decreases over age, although this depends on sex, and the type of exercise. Team-based competitive exercise activities appear more prone to decay than solitary, non-competitive activities. Tracking coefficients show voluntary exercise behaviour to be a moderate to highly stable behaviour, although this too depends on the age and domain of exercise. Comparing domains over the lifespan non-competitive and internally paced exercise proved to be the most stable domains, in particular in late adulthood. Stability decreases as the distance to follow-up increases, resulting in less carry-over from exercise at young ages to late adulthood. Team-based exercise in particular appears to be a poor predictor of the total volume of exercise behaviour in late adulthood. The varying results we found for age-effects, sex-effects, and the longitudinal tracking between the exercise domains substantiates the need to examine voluntary exercise behaviour as a function of age and sex, and split the activities across the various domains in favour of using a single score summarizing across all domains. For exercise interventionists, our results signal that the glass is half full. The substantial stability of this important health behaviour reinforces the existing evidence that exercise habits are hard to change, but at the same time suggests that any successful intervention that leads to the adoption of exercise habits stands a good chance to last.

## Additional files

Additional file 1: Descriptive table.pdf; Means and standard devaitions of MET-minutes across domains and ages. (PDF 100 kb)

Additional file 2: Within domain heatmaps.tif; Tracking coefficient, and sample size heatmaps of exercise domains, similar to Fig. 3 of the main manuscript. (TIF 22955 kb)

Additional file 3: Tracking coefficient descriptives.pdf; 95-percentile ranges, and medians of tracking coefficients. (PDF 116 kb)

Additional file 4: Tracking of Competitive exercise.tif; Tracking coefficients for volume of competitive exercise behaviour as a function of age at baseline. (TIF 2107 kb)

Additional file 5: Tracking of Non-competitive exercise.tif; Tracking coefficients for volume of non-competitive exercise behaviour as a function of age at baseline. (TIF 2353 kb)

Additional file 6: Tracking of Externally paced exercise.tif; Tracking coefficients for volume of externally paced exercise behaviour as a function of age at baseline. (TIF 2065 kb)

Additional file 7: Tracking of Internally paced exercise.tif; Tracking coefficients for volume of internally paced exercise behaviour as a function of age at baseline. (TIF 2389 kb)

Additional file 8: Tracking of Solitary exercise.tif; Tracking coefficients for volume of solitary exercise behaviour as a function of age at baseline. (TIF 2395 kb)

Additional file 9: Between-domain heatmaps.tif; Between-domain tracking coefficient heatmaps of exercise domains at baseline and total volume of exercise at follow-up. (TIF 23290 kb)

Additional file 10: Cross-domain tracking team-based exercise.tif; Tracking coefficients for volume of team-based exercise behaviour at baseline and total volume of exercise at follow-up, as a function of age at baseline. (TIF 2233 kb)

Additional file 11: Cross-domain tracking Solitary exercise.tif; Tracking coefficients for volume of solitary exercise behaviour at baseline and total volume of exercise at follow-up, as a function of age at baseline. (TIF 2448 kb)

Additional file 12: Cross-domain tracking Competitive exercise.tif; Tracking coefficients for volume of competitive exercise behaviour at baseline and total volume of exercise at follow-up, as a function of age at baseline. (TIF 2110 kb)

Additional file 13: Cross-domain tracking Non-competitive exercise.tif; Tracking coefficients for volume of non-competitive exercise behaviour at baseline and total volume of exercise at follow-up, as a function of age at baseline. (TIF 2407 kb)

Additional file 14: Cross-domain tracking Externally paced exercise.tif; Tracking coefficients for volume of externally paced exercise behaviour at baseline and total volume of exercise at follow-up, as a function of age at baseline. (TIF 2131 kb)

Additional file 15: Cross-domain tracking Internally paced exercise.tif; Tracking coefficients for volume of internally paced exercise behaviour at baseline and total volume of exercise at follow-up, as a function of age at baseline. (TIF 2369 kb)

## Abbreviations

MET: Metabolic Equivalent of Task; measure expressing the energy cost of a certain type of physical activity, or exercise. It is a measure relative to a person sitting at rest (1 MET), a higher MET score means more energy consumption
METmin: The amount of minutes a certain type of exercise is performed in one week multiplied by that type of exercise’s metabolic equivalent of task (MET) value. If multiple types are performed the METmin score is summed over all activities after multiplication
