# Supplementary material for: Tracking of voluntary exercise behaviour over the lifespan
Source: Int J Behav Nutr Phys Act. 2019 Feb 4;16:17. doi: 10.1186/s12966-019-0779-4 (PMC6360805; doi:10.1186/s12966-019-0779-4)
Supplement: Supplementary file 1 — Descriptive table.pdf; Means and standard devaitions of MET-minutes across domains and ages. (PDF 100 kb) [file 12966_2019_779_MOESM1_ESM.pdf]

	Total		Team		Sol		Comp		Ncomp		EP		IP	
**Age**	**M**	**SD**	**M**	**SD**	**M**	**SD**	**M**	**SD**	**M**	**SD**	**M**	**SD**	**M**	**SD**
8	527	517	170	374	357	409	232	423	294	366	230	421	194	336
10	766	667	383	596	382	464	487	628	279	386	486	636	124	304
12	872	798	427	704	445	556	556	742	316	486	549	741	157	396
14	984	908	528	820	456	606	663	865	321	514	659	870	160	394
16	1057	1047	540	857	518	764	665	912	392	676	658	906	225	529
18	1046	1186	479	891	567	915	584	956	462	825	578	960	317	702
20	912	1108	373	808	539	830	444	866	468	757	444	890	345	635
22	907	1066	314	737	594	853	391	807	516	800	391	826	413	701
24	921	1116	287	702	633	941	360	768	561	877	359	779	475	812
26	834	962	206	549	628	847	276	608	558	804	272	605	488	762
28	762	903	171	506	591	785	251	607	511	713	248	606	453	678
30	719	932	163	510	556	785	225	587	494	725	221	574	434	706
32	701	900	124	415	577	798	189	486	512	751	191	502	451	698
34	619	780	99	352	520	717	171	458	448	665	169	454	403	631
36	551	728	63	275	488	677	123	364	428	643	125	380	374	583
38	552	734	63	265	489	695	128	370	424	657	128	369	378	628
40	552	716	57	253	495	670	131	363	422	633	130	362	377	603
42	549	693	56	238	493	659	145	380	403	597	143	376	364	570
44	543	712	51	239	492	674	147	393	395	602	145	389	355	582
46	544	696	46	196	497	672	149	383	395	600	147	380	358	577
48	562	743	41	183	520	725	145	381	417	652	143	379	380	634
50	576	761	37	173	539	743	134	374	441	672	132	372	402	639
52	586	833	31	171	555	810	123	376	463	744	119	359	420	705
54	602	899	34	170	569	871	133	382	469	806	131	380	428	778
56	598	877	35	213	562	833	138	403	460	761	133	398	427	731
58	559	784	20	134	539	770	118	359	441	674	114	353	403	650
60	556	825	18	131	537	799	121	382	434	709	116	372	395	672
62	560	911	21	151	539	881	116	352	444	830	110	347	409	795
64	597	837	13	114	584	824	132	427	465	704	125	414	435	680
66	543	868	13	100	530	864	118	366	425	779	112	357	403	767
68	448	628	10	82	439	623	101	327	347	553	95	317	313	522
70	469	756	15	92	454	753	123	402	345	656	114	388	319	637
72	399	576	12	88	387	557	119	378	280	465	94	338	283	475
74	433	740	6	55	428	734	114	374	319	582	101	365	311	583
76	319	549	18	161	301	488	106	390	213	383	90	354	198	350
78	336	643	0	0	336	643	48	287	288	597	42	283	279	582
80	267	516	4	37	263	514	56	234	211	485	43	221	224	487
82	190	315	0	0	190	315	7	50	183	307	0	0	181	313

SUPPLEMENTARY TABLE 1. Mean, and standard deviation (SD) of MET-minutes in the 7 exercise dimensions over age. Comp: Competitive; Ncomp: non-competitive; EP: externally paced; IP: internally paced**.**
