# Supplementary material for: Tracking of voluntary exercise behaviour over the lifespan
Source: Int J Behav Nutr Phys Act. 2019 Feb 4;16:17. doi: 10.1186/s12966-019-0779-4 (PMC6360805; doi:10.1186/s12966-019-0779-4)
Supplement: Supplementary file 3 — Tracking coefficient descriptives.pdf; 95-percentile ranges, and medians of tracking coefficients. (PDF 116 kb) [file 12966_2019_779_MOESM3_ESM.pdf]

	2-year		8-year		14-year		20-year	
	**Range**	**Median**	**Range**	**Median**	**Range**	**Median**	**Range**	**Median**
**Within domain**								
**Total**								
Males	0.4-0.77	0.56	0.21-0.78	0.42	0.13-0.72	0.36	0.09-0.59	0.36
Females	0.37-0.71	0.53	0.23-0.64	0.39	0.2-0.65	0.32	0.26-0.5	0.35
**Team**								
Males	0.38-0.81	0.63	0.16-0.67	0.46	0.24-0.58	0.42	0.06-0.41	0.28
Females	0.52-0.98	0.7	0.14-0.79	0.48	0.15-1	0.65	0.03-0.72	0.23
**Competitive**								
Males	0.48-0.84	0.63	0.28-0.74	0.47	0.26-0.61	0.45	0.19-0.42	0.26
Females	0.53-0.83	0.69	0.36-0.7	0.49	0.26-0.91	0.45	0.21-0.66	0.37
**Externally paced**								
Males	0.47-0.88	0.64	0.29-0.72	0.51	0.28-0.6	0.45	0.21-0.43	0.26
Females	0.52-0.83	0.67	0.35-0.7	0.52	0.25-0.8	0.42	0.21-0.67	0.36
**Solitary**								
Males	0.24-0.77	0.54	0.11-0.8	0.4	0.08-0.82	0.42	0.18-0.75	0.4
Females	0.3-0.72	0.51	0.2-0.66	0.35	0.11-0.68	0.26	0.07-0.53	0.26
**Non-competitive**								
Males	0.26-0.78	0.56	0.06-0.93	0.37	0.05-0.92	0.36	0.07-0.81	0.43
Females	0.28-0.7	0.46	0.17-0.69	0.32	0.07-0.57	0.24	0.04-0.5	0.16
**Internally Paced**								
Males	0.22-0.75	0.55	0.12-0.87	0.39	0.04-0.84	0.34	-0.09-0.8	0.43
Females	0.26-0.77	0.44	0.15-0.73	0.33	0.06-0.42	0.23	0.01-0.48	0.1
**Between domains**								
**Team**								
Males	0.12-0.52	0.25	0.06-0.33	0.22	0.06-0.35	0.16	0.03-0.22	0.12
Females	0.01-0.5	0.24	0.03-0.38	0.22	0.03-0.35	0.17	0.03-0.4	0.19
**Competitive**								
Males	0.24-0.51	0.36	0.14-0.4	0.28	0.13-0.38	0.22	0.04-0.32	0.21
Females	0.27-0.49	0.33	0.18-0.42	0.29	0.11-0.4	0.2	0.13-0.35	0.24
**Externally paced**								
Males	0.23-0.52	0.37	0.14-0.39	0.28	0.13-0.37	0.26	-0.46-0.31	0.21
Females	0.26-0.47	0.33	0.18-0.42	0.29	0.11-0.41	0.2	0.13-0.37	0.24
**Solitary**								
Males	0.07-0.72	0.46	0.01-0.77	0.33	0.06-1.23	0.56	0.12-0.67	0.32
Females	0.14-0.7	0.48	0.03-0.64	0.31	0.03-0.66	0.28	0.02-0.52	0.26
**Non-competitive**								
Males	0.03-0.66	0.39	0.05-0.72	0.29	0.02-0.74	0.37	0.1-0.63	0.41
Females	0.11-0.58	0.4	0.03-0.71	0.26	0.03-0.46	0.2	0-0.44	0.2
**Internally Paced**								
Males	0.06-0.64	0.4	0.03-0.74	0.27	-0.01-0.84	0.41	-0.11-0.62	0.36
Females	0.04-0.57	0.37	0.02-0.76	0.25	0.08-0.44	0.2	0-0.41	0.22

SUPPLEMENTARY TABLE 2. 95-percentile ranges, and medians of tracking coefficients.
